# Cortical Areas Associated With Multisensory Integration Showing Altered Morphology and Functional Connectivity in Relation to Reduced Life Quality in Vestibular Migraine

**DOI:** 10.3389/fnhum.2021.717130

**Published:** 2021-08-16

**Authors:** Xia Zhe, Li Chen, Dongsheng Zhang, Min Tang, Jie Gao, Kai Ai, Weijun Liu, Xiaoyan Lei, Xiaoling Zhang

**Affiliations:** ^1^Department of MRI, Shaanxi Provincial People’s Hospital, Xi’an, China; ^2^Department of Neurology, Shaanxi Provincial People’s Hospital, Xi’an, China; ^3^Department of Clinical Science, Philips Healthcare, Xi’an, China; ^4^Consumables and Reagents Department, Shaanxi Provincial People’s Hospital, Xi’an, China

**Keywords:** vestibular migraine, cortical surface, surface-based morphometry, temporal lobe, parietal lobe, multisensory integration

## Abstract

**Background**: Increasing evidence suggests that the temporal and parietal lobes are associated with multisensory integration and vestibular migraine. However, temporal and parietal lobe structural and functional connectivity (FC) changes related to vestibular migraine need to be further investigated.

**Methods**: Twenty-five patients with vestibular migraine (VM) and 27 age- and sex- matched healthy controls participated in this study. Participants completed standardized questionnaires assessing migraine and vertigo-related clinical features. Cerebral cortex characteristics [i.e., thickness (CT), fractal dimension (FD), sulcus depth (SD), and the gyrification index (GI)] were evaluated using an automated Computational Anatomy Toolbox (CAT12). Regions with significant differences were used in a seed-based comparison of resting-state FC conducted with DPABI. The relationship between changes in cortical characteristics or FC and clinical features was also analyzed in the patients with VM.

**Results**: Relative to controls, patients with VM showed significantly thinner CT in the bilateral inferior temporal gyrus, left middle temporal gyrus, and the right superior parietal lobule. A shallower SD was observed in the right superior and inferior parietal lobule. FD and GI did not differ significantly between the two groups. A negative correlation was found between CT in the right inferior temporal gyrus, as well as the left middle temporal gyrus, and the Dizziness Handicap Inventory (DHI) score in VM patients. Furthermore, patients with VM exhibited weaker FC between the left inferior/middle temporal gyrus and the left medial superior frontal gyrus, supplementary motor area.

**Conclusion**: Our data revealed cortical structural and resting-state FC abnormalities associated with multisensory integration, contributing to a lower quality of life. These observations suggest a role for multisensory integration in patients with VM pathophysiology. Future research should focus on using a task-based fMRI to measure multisensory integration.

## Introduction

Vestibular migraine (VM) is considered to be the most common central cause of episodic vertigo, manifesting as moderately to severely intense vestibular symptoms and a migraine history. In 2012, the International Headache Society and the Ba’ra’ny Society proposed criteria for diagnosing VM as a disease entity (Lempert et al., 2012). An estimated 2.7% of adults suffer from vestibular migraines according to a recent population-based survey in the United States (Formeister et al., 2018). Women suffer from VM 2 to 3 times more frequently than do men (Neuhauser et al., 2001; Lempert and Neuhauser, 2009). VM is a disabling disorder that results in a significant burden on healthcare. Therefore, it is important to understand the pathophysiology of VM to help develop treatment plans for patients.

Previous studies indicate that the temporal lobe and the parietal lobe are associated with multisensory integration and vestibular processing (Obermann et al., 2014; Komeilipoor et al., 2017; Messina et al., 2017; Oh et al., 2018). Patients with VM have activity in the temporal and parietal lobes during VM attacks (Shin et al., 2014). Several voxel-based morphometric (VBM) studies have reported that patients with VM exhibit gray matter (GM) volume abnormalities in temporal lobe regions, including the superior temporal gyrus, middle temporal gyrus, and inferior temporal gyrus, as well as the parietal lobe (Obermann et al., 2014; Messina et al., 2017). Together, these findings strongly suggest that structural abnormalities in the temporal lobe are involved in multisensory integration, including visual, auditory, tactile, and vestibular processing (Beauchamp, 2005; Amedi et al., 2007). It is unclear, however, whether cerebral cortex characteristics alter multisensory integration and vestibular processing in brain areas during VM attacks. Surface-based morphometry (SBM) can focus on cortical structural characteristics, yielding more specific information about neurological development as well as changes in cortical function related to thinning of the cortex (Panizzon et al., 2009; Yotter et al., 2011a; Dahnke et al., 2013). Compared with VBM, SBM has been shown to be more sensitive and precise for detecting gray-matter atrophy, and it uses a completely automatic method, which provides the basis for projection-based thickness (PBT) measurement in order to obtain a local measure of GM within the cortex (Lemaitre et al., 2012; Dahnke et al., 2013). The SBM approach has been frequently used as a research method to assess cortical surface characteristics in migraine and other vestibular disorders (Komaromy et al., 2019; Nigro et al., 2019; Lai et al., 2020). However, no study to date has investigated the pattern of cerebral cortex characteristics and their changes in relation to the clinical features of VM.

Resting-state functional connectivity (FC) provides a powerful method to investigate the FC among brain regions, detecting the synchronized blood oxygen level-dependent (BOLD) signals from the seed region to the whole brain so as to locate highly correlated areas with similar characteristics (Xu et al., 2019; Niu et al., 2020). However, previous functional magnetic resonance imaging (fMRI) studies have used the amplitude of low-frequency fluctuation (ALFF) during external stimulation to assess functional changes in patients with VM. Russo et al. (2014) demonstrated that abnormal thalamic function is involved in central vestibular processing. Teggi et al. (2016) reported activation of brain areas related to integrating visual and vestibular cues in patients with VM undergoing fMRI during visual stimulation in vertigo-free periods. A fMRI study to observe treatment effectiveness found ALFF values in the left posterior cerebellum of patients with VM increased significantly after 1 month of vestibular rehabilitation training (Liu et al., 2020). Functional imaging demonstrated the cerebellum can improve vestibular functioning through a vestibular compensation mechanism. Recently, Wang et al. (2021) evaluated resting-state FC alterations in patients with VM during the interictal period. Although several fMRI studies have focused on ALFF or FC, no studies have explored FC alterations based on cortical structural abnormalities in patients with VM.

Given that previous findings have shown that changes in the gray-matter volume of the temporal and parietal lobes are related to multisensory vestibular processing in VM (Obermann et al., 2014; Messina et al., 2017; Zhe et al., 2020), we hypothesized that alterations in cerebral cortex characteristics in patients with VM could be located in brain regions associated with multisensory vestibular processing. And we also hypothesized that changes in cortical regions were accompanied by changes in FC. Therefore, in the current study, we used a whole-brain SBM technique to evaluate cortical surface characteristics in VM patients, during an interictal period, compared with healthy controls. Furthermore, we used seed-based FC to investigate whether cortical regions with structural abnormalities also exhibit FC alterations in a patient with VM. Additionally, we assessed the relationships between brain morphological or FC changes and clinical parameters.

## Materials and Methods

### Subjects

Patients were recruited from the vertigo and dizziness outpatient service center of the Shaanxi Provincial People’s Hospital in China between January 2016 and October 2020, who were diagnosed with VM by a neurologist based on the International Classification of Headache Disorder 3rd edition criteria (Lempert et al., 2012). Twenty-five right-handed patients with VM (21 without aura and four with aura) and 27 healthy controls participated in this study. Patients were excluded if they had a history of other neurologic, psychiatric, audiovestibular, or systemic disorders. All patients in a symptom-free interval underwent a routine neurologic and neuro-otological examination, as well as MRI scanning, which were performed on the same day. No peripheral vestibular dysfunction was found in videonystagmography (VNG) recordings. The clinical symptoms of each patient were assessed using a Visual Analog Scale (VAS; 0 = no pain; 10 = worst possible pain), the Migraine Disability Assessment Scale (MIDAS), the Headache Impact Test-6 (HIT-6), and the Dizziness Handicap Inventory (DHI) using face-to-face interviews with a standardized questionnaire and questions (Sauro et al., 2010; Hawker et al., 2011; Balci et al., 2018). Eight of the patients with VM were treated with migraine-preventive medications and nonsteroidal analgesics. Most patients (*n* = 17) did not take any medication regularly.

The 27 age-, sex- and handedness-matched healthy controls were from the community. The exclusion criteria were: migraine; chronic pain; previous vestibular neuritis; Meniere’s disease; secondary somatoform vertigo; drug abuse; neurologic, mental or systemic disorders; ischemic or hemorrhagic stroke; or severe head trauma. All subjects had no structural abnormalities or white matter (WM) lesions in T2-weighted or FLAIR imaging. This study was approved by the Ethics Committee of the Shaanxi Provincial People’s Hospital. All participants provided written informed consent before entering the study.

### Imaging Data Acquisition

All the images were obtained using a 3.0 T Philips Ingenia scanner with a 16-channel phased-array head coil. A high-resolution three-dimensional (3D) magnetization-prepared rapid-acquisition gradient echo (MPRAGE) T1-weighted (T1w) sequence covering the whole brain (332 sagittal slices) was collected. The acquisition parameters were: repetition time (TR) = 1,900 ms; echo time (TE) = 2.26 ms; inversion time (TI) = 900 ms; flip angle (FA) = 9°; matrix = 256 × 256; field of view = 220 × 220 mm; and 1.00 mm slice thickness with no interslice gap. Resting-state functional BOLD images were scanned using gradient echo-planar imaging with the following parameters: repetition time = 2,000 s; echo time = 30 ms; slices = 34; slice thickness = 4 mm; slice gap = 0 mm; field of view = 230 × 230 mm; matrix = 128 × 128; flip angle = 90°; and 200 volumes. For the resting-state scan, all subjects were asked to keep their eyes closed and their minds calm, and to stay awake throughout the scan. After the scan, subjects were asked whether or not they remained awake during the entire procedure.

### Image Processing

Structural images were processed using CAT12^1^ and SPM12 run in MATLAB R2014b (The MathWorks, Inc.). CAT12 provides a volume-based method for estimating regional thickness (CT) without extensive reconstruction of the cortical surface and has been shown to be a fast and reliable alternative to FreeSurfer (Paul et al., 2017; Seiger et al., 2018). Moreover, CAT12 is a fully automated method that allows the measurement of the whole brain cortical surface. For each participant, the processing pipeline included bias-field, noise removal, skull stripping, and segmentation into GM, WM, and cerebrospinal fluid (CSF). The images were finally normalized to MNI space, which uses diffeomorphic anatomical registration using exponentiated Lie algebra (DARTEL) to a 1.5 mm isotropic adult template (Ashburner, 2007). Here, the CT evaluation and reconstruction of the central surface were performed in one step, based on the PBT method (Dahnke et al., 2013). Importantly, the PBT allows the appropriate handing of partial volume information, sulcal blurring, and sulcal asymmetries without explicit sulcus reconstruction (Dahnke et al., 2013). After the initial surface reconstruction, topology correction (Yotter et al., 2011b), spherical mapping (Yotter et al., 2011a), and spherical registration were conducted. In addition, CAT12 allows the estimation of other morphological indices of fractal dimension (FD), sulcus depth (SD), and gyrification index (GI), which were also calculated for each participant with default parameter settings. The calculation of CT, FD, SD, and GI was performed in subject native surface space. The images of cerebral cortex characteristics were checked for homogeneity. As all the images had high correlation values (>0.85), none of them had to be discarded. Finally, the CT images were smoothed using a Gaussian kernel with a full width at half maximum (FWHM) of 15 mm, and three other surface parameters were smoothened with an isotropic 20 mm FWHM Gaussian kernel.

All functional images were preprocessed using Data Processing and Analysis for Brain Imaging 3.0^2^, which is based on Statistical Parametric Mapping 12^3^. First, the first 10 volumes were removed to allow subjects to adapt to the magnetic field. Second, slice timing correction was performed to correct for the inter-slice time delay within each volume. Third, head motion >1.5 mm and translation >1.5° of rotation in any direction were excluded. Images were spatially normalized into MNI space using a standard EPI template provided by SPM12 and resliced into a voxel size of 3 × 3 × 3 mm. Finally, data were spatially smoothed using a 6-mm FWHM Gaussian kernel.

Seed-based FC analysis was performed with seeds from the SBM findings. Seeds were defined as 3-mm-radius spheres centered on the peak voxel for the CT and SD clusters showing between-group differences. The averaged time-course of each seed area was extracted, and Pearson’s correlation (*r*) was used to calculate the FC between the extracted time-courses and the time-courses of the entire brain in a voxel-wise manner. The individual *r*-maps were normalized to Z-maps using Fisher’s Z-transformation.

### Statistical Analysis

#### Demographic and Clinical Data

A two-sample *t*-test was used to estimate the differences in age, sex, and years of education between the VM and healthy control groups. The statistical significance level was set at *P* < 0.05. These statistical analyses were performed using the SPSS software package (version 22.0).

#### Cortical Surface Characteristics Analysis

Cortical surface characteristics were compared between the VM patients and healthy controls using two-sample *t*-tests in CAT12 with age and sex as covariates. Family-wise error (FWE) correction was performed to correct for multiple comparisons; *P* < 0.05 was considered statistically significant. Then, the surviving clusters were reported. Finally, based on the Desikan–Killiany (DK40) atlas (Desikan et al., 2006), we extracted the mean cortical surface characteristics (CT, SD, GI, and FD) from the above mentioned significant clusters. Partial correlations adjusted for age and sex were used to analyze differences between the cortical surface characteristics of these altered regions and clinical indices (including the VAS score, disease duration, attack frequency, MIDAS score, HIT-6 score, and DHI score). The significance threshold was set at *P* < 0.05.

#### Seed-Based FC Analysis

A comparison of FC between groups was performed using a two-sample *t*-test within DPABI, with age and sex as covariates. Correction for multiple comparisons was performed using a Gaussian random field at *P* < 0.05 (voxel *P* < 0.001). Then, the surviving clusters were reported.

Finally, we extracted the average *Z*-values for each region with significant differences and performed a partial correlation analysis with patients’ clinical parameters using SPSS 22.0, controlling for age and sex. The significance threshold was set at *P* < 0.05.

## Results

### Demographic and Clinical Data

There were no significant differences between the VM patients and healthy controls in age, sex, or years of education. The results are summarized in Table 1. VM patients suffered from a moderate and severe migraine burden with a mean VAS score of 4.74 ± 2.75, mean HIT-6 score of 51.56 ± 19.94, and mean MIDAS score of 54.33 ± 53.13. Their scores on the vertigo scale were moderate with a mean DHI score of 48.93 ± 16.43.

**Table 1:** Demographic and clinical characteristics of patients.

Characteristics	VM (*n* = 27) Mean ± SD	HC (*n* = 25) Mean ± SD	*P* value
Sex (female/male)	27/4	25/4	0.91
Age (years)	38.22 ± 10.58	37.28 ± 11.45	0.76
Education (years)	13.89 ± 3.61	14.40 ± 2.48	0.56
Disease duration (years)	9.15 ± 7.58		
Headache frequency (number)	6.67 ± 4.93		
VAS	4.74 ± 2.75		
MIDAS	54.33 ± 53.13		
HIT-6	51.56 ± 19.94		
DHI	48.93 ± 16.43		

*Note: VM, vestibular migraine; HC, healthy control; VAS, Visual Analog Scale (0 = no pain, 10 = worst possible pain); MIDAS, Migraine Disability Assessment Scale; HIT-6, Headache Impact Test-6; DHI, Dizziness Handicap Inventory*.

### Cortical Surface Characteristics Results

**Figure 1:**
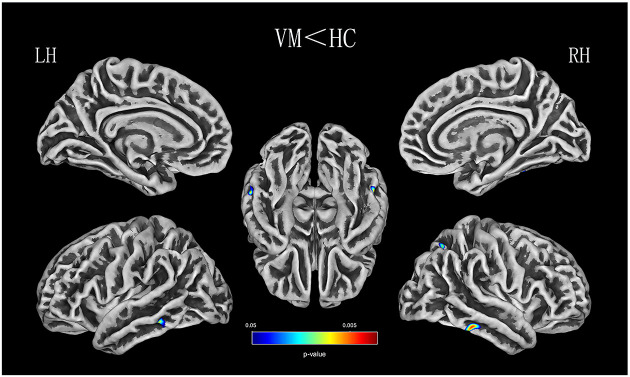
CT (thickness) analysis results of patients with vestibular migraine (VM) compared with healthy controls [*P* < 0.05, family-wise error (FWE)-corrected].

**Figure 2:**
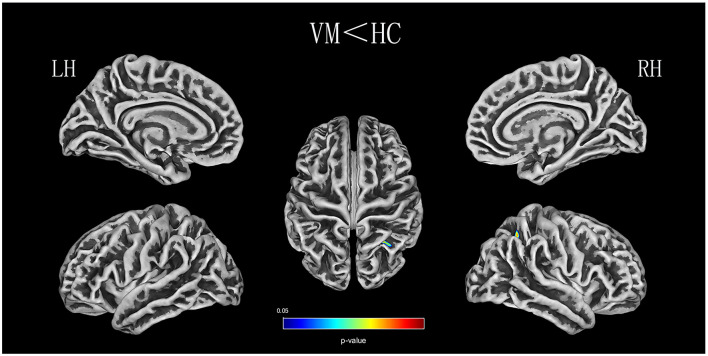
SD (sulcus depth) analysis results of patients with VM compared with healthy controls (*P* < 0.05, FWE-corrected).

**Figure 3:**
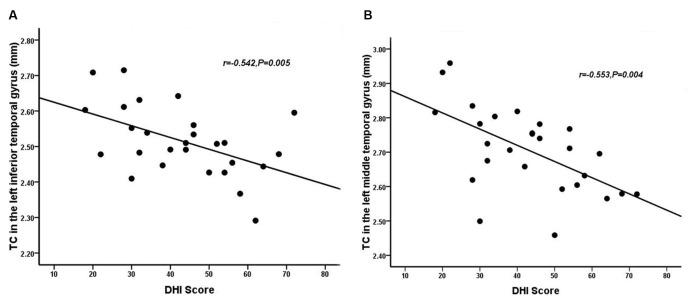
Correlation between the CT of the inferior **(A)** and middle temporal gyrus **(B)** and dizziness handicap inventory (DHI) score in patients with VM (*P* < 0.05).

Relative to healthy comparison subjects, the VM patients showed significantly thinner CT in the bilateral inferior temporal gyrus, left middle temporal gyrus, and right superior parietal lobule (Table 2, Figure 1). Reduced SD was found in the right superior and inferior parietal lobule (Table 3, Figure 2). There was no significant intergroup difference for surface parameters GI and FD. In the VM patients, a significant negative correlation was found between DHI scores and the CT of the right inferior temporal gyrus (*r* = −0.542; *P* = 0.005; Figure 3A) and left middle temporal gyrus (*r* = −0.553; *P* = 0.004; Figure 3B). No correlation was found between abnormal SD and disease duration, attack frequency, VAS score, MIDAS score, HIT-6 score, or DHI score in the VM patients.

**Table 2:** Decreased CT in various brain regions in patients with VM.

	Brain regions	Peak MNI			Cluster voxels	*T*	*Z*	*P*
R	Inferior temporal gyrus	63	−36	−22	149	5.38	4.74	0.000
	Superior parietal lobule	24	−63	50	68	5.06	4.60	0.000
L	Inferior temporal gyrus	−56	−38	−14	102	5.19	4.51	0.000
	Middle temporal gyrus	−56	−38	−14	102	5.19	4.51	0.000

*Note: MNI, Montreal Neurological Institute; R, right; L, Left; CT, thickness; VM, vestibular migraine*.

**Table 3:** Decreased SD in various brain regions in patients with VM.

	Brain regions	Peak MNI			Cluster voxels	*T*	*Z*	*P*
R	Superior parietal lobule	28	−51	49	382	5.02	4.48	0.000
	Inferior parietal lobule	32	−60	47	382	4.91	4.39	0.000

*Note: MNI, Montreal Neurological Institute; R, right; SD, sulcus depth*.

### Seed-Based FC Results

**Figure 4:**
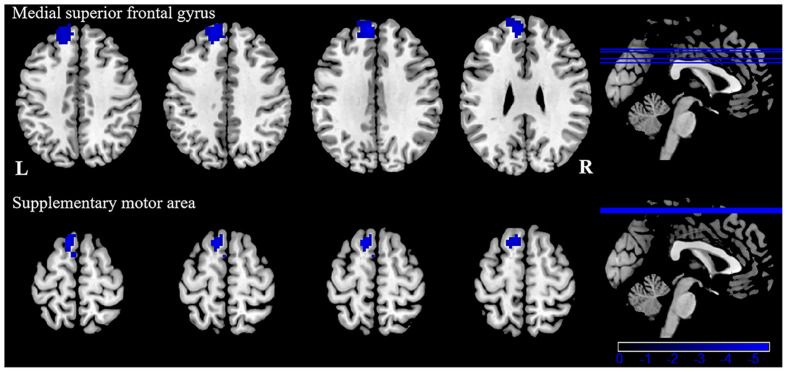
Functional connectivity (FC) analysis results for patients with VM compared with healthy controls (*P* < 0.05, GRF-corrected).

Patients with VM showed significantly weaker FC between the left inferior/middle temporal gyrus and the left superior frontal gyrus, supplementary motor area (Table 4, Figure 4). There were no significant group differences in FC with other seed regions (right inferior temporal gyrus, right superior, and inferior parietal lobule). No significant correlation was observed between FC alterations and clinical characteristics in patients with VM.

**Table 4:** Abnormal functional connectivity of the left inferior/middle temporal gyrus in patients with VM.

	Seed points		Brain region	BA	Peak MNI			Cluster voxels	*T*
L	Inferior/middle temporal gyrus	L	Medial superior frontal gyrus	9	−9	51	33	248	−5.54
		L	Supplementary motor area	6	−9	18	69	75	−5.12

*Note: MNI, Montreal Neurological Institute; R, right*.

## Discussion

As far as we know, our study is the first to directly investigate cortical surface characteristics and FC changes in patients with VM and healthy controls, as well as associations between cortical surface characteristics or FC and clinical variables. Compared with healthy controls, we found that patients with VM had decreased CT and SD in certain areas, including multisensory integration and vestibular processing regions. Additionally, we found that DHI scores and CT were significantly correlated in the right inferior temporal gyrus and the left middle temporal gyrus. Using the clusters derived from the SBM analysis as seed regions, we found significantly weaker FC between the left inferior/middle temporal gyrus and the left medial superior frontal gyrus, supplementary motor area. Our data confirmed our hypothesis that VM patients have abnormalities in cortical surface characteristics related to multisensory vestibular processing and that changes in cortical regions are accompanied by changes in FC.

The temporal lobe has been recognized as a region associated with multisensory integration, which involves auditory, olfactory, vestibular, and visual senses and the perception of spoken and written language (Kiernan, 2012). A number of studies on patients with migraine have found that CT was thinning in the temporal lobe, suggesting that the temporal lobe plays an important role in the regulation of pain (Coppola et al., 2017; Jia and Yu, 2017). Several studies have demonstrated greater interregional CT correlations in patients with migraine, specifically over temporal regions (Chong et al., 2020). Schwedt et al. (2015) found that temporal pole correlations distinguished groups of migraine patients from healthy controls. An fMRI study of 12 right-handed patients with VM, which used cold caloric stimulation, found a typical pattern of BOLD signals in temporal-parietal areas in the interictal interval, including patients with migraine without aura and healthy controls (Russo et al., 2014). A recent functional imaging study of two patients reported that the metabolism of the temporoparietal-insular areas increased during a VM attack (Fasold et al., 2002). These results suggest that some modification of structural covariance patterns in the temporal lobe is involved in pain-processing and multisensory integration (Moulton et al., 2011). The VM patients in that study also showed reduced CT in multiple areas of the temporal lobe, including the inferior and middle gyrus, compared with healthy controls, which is in line with the findings of several previous studies. Obermann et al. (2014) found that gray-matter volume was decreased in the inferior temporal gyrus, middle temporal gyrus, and the superior temporal gyrus, the middle cingulate, dorsolateral prefrontal, insular, parietal, and occipital cortices (Obermann et al., 2014). These structurally abnormal brain areas in patients with VM are involved in multisensory vestibular control, as well as pain processing and central vestibular compensation. In contrast, a recent VBM study found an increase in the temporal lobe, frontal lobe, and occipital lobe in VM patients compared with healthy controls (Messina et al., 2017). These inconsistent findings might be due to differences in sample size, attack frequency, medication status, and data acquisition and processing in the various studies.

The inferior temporal gyrus is related to visual processing (Naito et al., 2003). There is some evidence that decreased CT in the inferior temporal gyrus might contribute to abnormalities in multisensory integration of visual processing, such as amplifying vision (photophobia), hearing (phonophobia), or olfactory stimuli, which may induce an attack of VM. Repeated VM attacks over time that seem to lead to an alteration of multisensory integration of visual processing structures may provide an explanation as to why most VM patients have increased sensitivity to visual, auditory, and olfactory stimuli during VM attacks. Previous studies have also suggested that the middle temporal gyrus plays a key role in interconnecting with other multisensory cortical areas, and it is deemed to form a multisensory integrative network (Helmchen et al., 2014). The middle temporal gyrus, inferior temporal gyrus, and superior temporal gyrus, and the lateral temporal lobe play a role in the underlying connection between migraine and the vestibular system (Rocca et al., 2006; Schwedt et al., 2013; Helmchen et al., 2014). The middle temporal gyrus belongs to the temporal perisylvian vestibular cortex, which is particularly sensitive for dizziness (Kahane et al., 2010). Furthermore, CT in the inferior and middle temporal gyrus is negatively correlated with the severity of vertigo in VM patients. The DHI was used to evaluate the self-perceived handicapping effects of dizziness, which is related to the physical, emotional, and functional aspects of patients. Vertigo attacks result in subjective spatial orientation errors, surrounding environment spiraling around, and complaining of imbalance in patients. That may lead to patients who usually dare not to attempt daily activities, and experience obvious anxiety and depression which reflect the degree of vertigo. In the present study, a mean DHI score of 48.93 points was obtained. In patients with VM, as the DHI score increased, there was a decrease in life quality scales showing moderate disability in DHI. However, we did not evaluate symptoms of depression and anxiety. Thus, it is not clear whether anxiety or depression is associated with DHI in patients with VM. Future studies should clarify this. Correlations revealing a CT decrease in the temporal lobe was associated with an increased subjective intensity of vertigo in VM, which indicated that the temporal lobe is involved in the pathophysiology of patients with VM and is associated with the daily life of the patient. Therefore, CT reduction in the inferior and middle temporal gyrus is a potentially valuable morphological characteristic, which might result in central vestibular syndromes that manifest along with vertigo and dizziness. Based on all of the above discussion, our results indicate that long–term and high-frequency headaches and vertigo attacks may lead to reduced CT in multisensory integrative and vestibular processing areas in VM, reflecting abnormal brain structure due to the effects of brain disease. This has profound implications for our understanding of multisensory integrative networks in patients with VM.

The second finding of the current study is decreased CT in the superior parietal lobule. Furthermore, we found lower SD in the inferior and superior parietal lobule in patients with VM compared with healthy controls. Other studies proposed that the parietal lobule is chiefly involved in discriminating sensory features of pain (Hofbauer et al., 2001; Oshiro et al., 2007, 2009). The superior parietal lobule, as a part of the parietofrontal network, has been found to be related to the perceptual matrix of pain (Garcia-Larrea and Peyron, 2013). It also contains major parts of the sensory cortex that are involved in spatial orientation and sensory information processing and interpretation (Kamali et al., 2014). Studies have confirmed that the inferior parietal lobe belongs to part of the multisensory vestibular cortical network involved in pain and vestibular processing (Dieterich and Brandt, 2008). The parietal lobule has been implicated in VM, and some VBM studies on patients with VM have reported a lower gray-matter volume in the parietal lobes of such patients compared with controls (Obermann et al., 2014; Zhe et al., 2020). A recent fMRI study of two VM patients reported increased activity in the inferior parietal lobule during visual stimulation in a vertigo-free period (Teggi et al., 2016). Correlation analysis also revealed that decreased gray-matter volume in the parietal lobe is associated with illness duration and headache intensity in patients with VM (Obermann et al., 2014). These studies indicate that cortical abnormalities of the parietal lobe are involved in nociception and multisensory vestibular control. Our findings in VM patients implicate the parietal lobe in the modulation of pain perception and dysfunction of sensory integration.

In order to assess if these cortical structural abnormalities also exhibited FC alterations, we performed a resting-state FC study. Our results showed decreased FC between the left inferior/middle temporal gyrus and left medial superior frontal gyrus supplementary motor area in patients with VM, compared to healthy controls. The medial superior frontal gyrus is not only involved in emotional responses and feelings of pain, but also in memory, attention responses, and cognitive reactions related to pain (Bluhm et al., 2007). The study confirmed that the superior frontal gyrus is involved in the integration of somatosensory and vestibular information (Klingner et al., 2016). Our research indicates that the endogenous analgesic mechanism of VM patients is adjusted because some long–term migraine and vertigo attacks occur, altering the emotional response to pain, or reducing pain perception and cognition, which can reduce the input of pain signals. Anatomically, the SMA is located in the dorsomedial frontal cortex, which is involved in executive control (Aron and Roldrack, 2006; Li et al., 2006), pain anticipation (Koyama et al., 2005; Rainville et al., 2009), and an affective component of pain (Apkarian et al., 2005; Geha et al., 2008). Our results indicate that the FC decrease between the left inferior/middle temporal gyrus and left supplementary motor area may be related to deficits in affective pain modulation and affective pain response inhibition. In addition, the SMA is associated with auditory processing (Lima et al., 2016). Phonophobia is reported in about half of patients with VM during a vertigo attack (Neuhauser et al., 2006). Exposure to noise may cause generalized discomfort and increase the pain and vertigo of the patients with VM. Recurrent VM attacks may ultimately result in FC alterations associated with auditory processing. Thus, the FC changes may serve as a possible explanation for phonophobia when vertigo occurs (Wei et al., 2020), which provides a new clue for therapy for this syndrome.

The present study has several limitations. First, our study was conducted with a relatively small sample. Our study should have included a larger sample that was more representative of a pathological population, which would help to assure greater reproducibility of its results. Second, subgroup analyses of migraine (migraine without aura and migraine with aura) were not performed. To better elucidate the cortical morphological difference between them, future studies should compare the two types of VM, and it would be valuable to recruit a larger sample size to be able to divide participants into the subgroups “migraine without aura” and “migraine with aura.” Third, we did not examine subcortical and brain stem structures in this study; therefore, future studies need to be designed to include both. Fourth, because the sample size was relatively small, the correlation analysis was not strictly conducted with Bonferroni corrections. Fifth, we did not use an experimental task to measure multisensory integration. Finally, we did not evaluate symptoms of depression and anxiety, although previous research has reported that patients with VM have high levels of depression and anxiety (Kim et al., 2016). The burden of symptomatology can affect cortical morphology. Assessment of depression and anxiety scores should be performed in future studies.

## Conclusion

In conclusion, we evaluated cortical structural and FC alterations in patients with VM using SBM and resting-state FC analyses, compared with healthy controls. CT and SD abnormalities were detected in the temporal lobe and parietal lobe. Furthermore, patients with VM displayed decreased FC between the left inferior/middle temporal gyrus and the left superior frontal gyrus, supplementary motor area. These regions are known to be involved in multisensory integration, vestibular processing, and pain modulation, contributing to a lower quality of life. These findings will promote our understanding of the underlying mechanism of VM, but so far an experimental task to measure multisensory integration in patients with VM has not been used. Future studies should identify brain areas associated with multisensory integration using a task-based fMRI. Moreover, further studies focusing on anxiety and depression are needed, which are bound to shed light on emotional states in patients with VM.

## Data Availability Statement

The original contributions presented in the study are included in the article, further inquiries can be directed to the corresponding author.

## Ethics Statement

The studies involving human participants were reviewed and approved by the Ethics Committee of the Shaanxi Provincial People’s Hospital. The patients/participants provided their written informed consent to participate in this study.

## Author Contributions

XZhe drafted the manuscript, designed the experiment, and performed the statistical analysis. LC undertook clinical parameters assessments. MT, JG, XL, and DZ collected the data. KA and WL provided technical support. XZha made study supervision or coordination. All authors contributed to the article and approved the submitted version.

## Conflict of Interest

Author KA was employed by the company Philips Healthcare. The remaining authors declare that the research was conducted in the absence of any commercial or financial relationships that could be construed as a potential conflict of interest.

## Publisher’s Note

All claims expressed in this article are solely those of the authors and do not necessarily represent those of their affiliated organizations, or those of the publisher, the editors and the reviewers. Any product that may be evaluated in this article, or claim that may be made by its manufacturer, is not guaranteed or endorsed by the publisher.

## Footnotes

^1^ http://dbm.neuro.uni-jena.de/cat12

^2^ http://rfmri.org/dpabi

^3^ http://www.fil.ion.ucl.ac.uk/spm
